# Supplementary material for: Immunoinformatics approach to engineer a multi-epitope vaccine against SdrG in skin commensal Staphylococcus epidermidis
Source: PLoS One. 2026 Mar 5;21(3):e0327534. doi: 10.1371/journal.pone.0327534 (PMC12962517; doi:10.1371/journal.pone.0327534)
Supplement: S1 File — (DOCX) [file pone.0327534.s001.docx]

**Supplementary Tables**

**Supplementary Table S1:** Antigenicity prediction for virulence proteins of *S. epidermis* by VaxiJen server. The Bacterial subcellular location of the protein was assessed by PsortB.

| Serial No | Proteins name | Accession number | Vaxijen score | PsortB (score) |
| --- | --- | --- | --- | --- |
| 01. | Accumulation-associated protein (Aap) | WP_285335572.1 | 0.7593 | Cell wall (10.00) |
| 02. | Serine-Aspartate Repeat Protein G (SdrG) | WP_002470795.1 | 0.7765 | Cell wall (10.00) |
| 03. | Extracellular matrix-binding protein (Embp) | WKF45240.1 | 0.5361 | Extracellular (2.50) |
| 04. | Poly-β-6, 1-N-acetyl-D-glucosamine (PNAG) | P75905 | 0.6026 | Cytoplasmic Membrane (10.00) |
| 05. | Serine protease Esp | AAO05142.1 | 0.5142 | Extracellular (10.00) |
| 06. | Cysteine protease EcpA | BEJ45134.1 | 0.5965 | Extracellular (8.19) |
| 07. | Metalloprotease SepA | Q5HKU0 | 0.6875 | Extracellular (9.98) |

**Supplementary Table S2: The predicted B cell epitopes of SrdG.**

**ABCpred (Predicted Linear Epitopes)**

Sequence Start positiion Score

ERMIDISLLFGLGHNE 78 0.97

SGEIIATGTYDNTNKQ 396 0.97

TPEGYTPTLKHSGTNP 703 0.96

DSDSDSDSDSDSGLDN 894 0.94

NGNIISTTTDENGKYQ 782 0.93

TVEYQKPNENRTNLQS 464 0.93

SKIIESNTESNKEENT 237 0.93

HVTITDHDDFSIDNGY 837 0.92

TQTTTDSGDDDEQDAD 817 0.92

DFSIDNGYYDDDSDSD 845 0.91

TYKIGDYVWEDVDKDG 637 0.91

SNRIYDYSEYEDVTND 539 0.91

TSYIDKSKVPNNNTKL 433 0.91

QIKKEETNSNDAIENR 142 0.91

DSDSDSDSDSDSDSDS 886 0.90

DSDSDSDSDSDSDSDS 886 0.90

GFLAGSPRECRSRSTA 50 0.89

PARTATEREPEATCNT 22 0.89

ASIQTSDNEENSRVSD 218 0.89

DSDSDSGLDNSSDKNT 900 0.88

INGQDDMIDSGFYQTP 731 0.88

DYTIQQTVTMQTTINE 590 0.88

RECRSRSTAPHYLCCC 57 0.88

KVKSGDTMTVNIDKNT 363 0.88

DGIIKAHDAENLIYDV 341 0.88

KDGIQNTNDNEKLSNV 650 0.87

SGQGQGDLPPEKTYKI 625 0.87

PSSVESSNSSMDTAQP 194 0.87

SSDKNTKDKLDTGANE 910 0.86

NYVWYDTNKDGIQGDD 752 0.86

SGFYQTPKYSLGNYVW 740 0.86

LKHSGTNPALDSEGNS 711 0.86

TSKSVRTDEEGKYQFD 675 0.86

EDSITSQPSSYKNIDK 261 0.86

GVKVTLKDENGNIIST 773 0.85

PYIIKVISKYDPNKDD 575 0.85

DVTNDDYAQLGNNNDV 550 0.85

DQSITEGYDDSDGIIK 330 0.85

REPEATCNTAININGP 29 0.85

QTIYINPLRYSAKETN 493 0.84

DVEYKTALSSVNKTIT 449 0.84

QITYTFTDYVDKYENI 411 0.84

LGALLLGKRRKNRKNK 941 0.83

IQGDDEKGISGVKVTL 763 0.83

ENTIEQPNKVREDSIT 250 0.83

DSTIIKVYKVGNQNLP 522 0.82

TFEVDDKVKSGDTMTV 357 0.82

YIVHFDKPSGMTQTTT 806 0.81

NVNISGNGDEGSIDDS 508 0.81

QPSHTTINSEASIQTS 208 0.81

KDSNMDDELSDSNDQS 105 0.81

AEENTVQDVKDSNMDD 96 0.8

DNTIAFSTSSGQGQGD 616 0.8

MQTTINEYTGEFRTAS 599 0.8

PLRYSAKETNVNISGN 499 0.8

MFTNIDTKNHTVEQTI 480 0.8

TGTYDNTNKQITYTFT 402 0.8

RPLSTTSAQPSSKRVT 296 0.8

SQSINTDDDNQIKKEE 132 0.8

LGHNEAKAEENTVQDV 89 0.79

LCCCSEPIDERMIDIS 69 0.79

YENIKAHLKLTSYIDK 423 0.79

CNTAININGPRTEING 35 0.79

TFLQKTPQDNTQLKEE 174 0.79

NGPRTEINGFLAGSPR 42 0.78

DDEQDADGEEVHVTIT 826 0.77

GLTKITFETPEGYTPT 695 0.77

TGEFRTASYDNTIAFS 607 0.77

TVNIDKNTVPSDLTDS 371 0.77

NSVWVTINGQDDMIDS 725 0.76

VGNQNLPDSNRIYDYS 531 0.76

KLDTGANEDHDSKGTL 918 0.75

NAMEFLLSERINEASP 7 0.75

TDYVDKYENIKAHLKL 417 0.75

VTLTYPDGTSKSVRTD 667 0.74

QLAAEGSNVNHLIKVT 314 0.74

YDPNKDDYTIQQTVTM 584 0.73

AQLGNNNDVNINFGNI 557 0.73

PKIKDNSGEIIATGTY 390 0.73

TESNKEENTIEQPNKV 244 0.73

NDVINNSQSINTDDDN 126 0.73

QSSNEEKNDVINNSQS 119 0.73

EGKYQFDGLKNGLTKI 684 0.72

PSDLTDSFAIPKIKDN 380 0.72

YKNIDKISNQDELLNL 271 0.72

ELSDSNDQSSNEEKND 112 0.72

YVWEDVDKDGIQNTND 643 0.7

ELLNLPINEYENKVRP 282 0.7

SERINEASPARTATER 14 0.7

TTTDENGKYQFDNLNS 788 0.69

KVPNNNTKLDVEYKTA 440 0.68

NLIYDVTFEVDDKVKS 351 0.68

AQPSSKRVTVNQLAAE 303 0.68

NDAIENRSKDITQSTT 151 0.68

GKYQFDNLNSGNYIVH 794 0.67

FGNIDSPYIIKVISKY 569 0.67

EYENKVRPLSTTSAQP 290 0.67

KGTLLGALFAGLGALL 930 0.61

NVDENEATFLQKTPQD 167 0.6

### ****Predicted Linear B-Cell Epitopes in SdrG Protein (BepiPred-2.0)****

| **Epitope ID** | **Start-End Positions** | **Sequence** | **Length (aa)** | **Average Score** | **Remarks** |
| --- | --- | --- | --- | --- | --- |
| E1 | 5–266 | LGHNEAKAEE...DAENL | 262 | 0.612 | Longest epitope; high antigenicity |
| E2 | 273–337 | FEVDDKVKSG...DKYEN | 65 | 0.589 | Discontinuous motifs |
| E3 | 334–336 | DYV | 3 | 0.536 | Solvent-exposed peak |
| E4 | 415–417 | PLR | 3 | 0.533 | Overlaps with DiscoTope-2.0 |
| E5 | 500–504 | YDPNK | 5 | 0.543 | Potential antibody-binding site |
| E6 | 520–525 | NEYTGE | 6 | 0.627 | Highest average score |
| E7 | 673–676 | TNKD | 4 | 0.554 | Validated by ABCpred |

| Emini surface accessibility Prediction (Predicted Linear Epitopes) | | |
| --- | --- | --- |
| Sequence | Sequence | Sequence |
| DSGDDDEQDAD | KTPQDNTQL | NTNDNEK |
| DSNRIY | KYDPNKDDYT | SDNEEN |
| EYQKPNENRTN | NEAKAE | SNDQSSNEEK |
| GYYDDDSDSDSDSDSDSDSDSDSDSDSDSDSDSDSDSDSDSDSDSDSDSDSD | NEYENK | SSDKNTKDK |
| KSVRTDEEGKYQ | NRSKDI | TDDDNQIKKEETNS |
| TTDENGKYQ | YSEYEDVTNDD | YVDKYE |
| TYDNTNKQI |  |  |

**Ellipro (Predicted Linear Epitopes)**

| Sequence | Score | Sequence | Score |
| --- | --- | --- | --- |
| DDFSIDNGYYDDDSDSDSDSDSDSDDSDSDSDSDSDSDSDSDSDSDSDSDSDSDSDSDSDSDSDSDSDSGLDNSSDKNTKDKLP | 0.846 | MIKKNNLLTKKKPIANKSNKYAIRKF | 0.803 |
| KDSNMDDELSDSNDQSSNEEKNDVINNSQSINTDDDNQIKKEETNSNDAIENRSKDITQSTTNVDENEATFLQKTPQDNTQLKEEVVKEPSSVESSNSSMDTAQQPSHTTINSEASIQTSDNEENSRVSDFANSKIIESNTESNKEENTIEQPNKVREDSITSQPSSYKNIDEK | 0.791 | LLGKRRKNRKNKN | 0.766 |
| EYENKVRPLSTTSAQPSSKRV | 0.68 | IVHFDKPSGMTQTTTDSGDDDEQDADGEEVHVT | 0.68 |
| SLGNYVWYDTNKDGIQGDDEKGISGVKVTLKDENGNIISTTTTDENGKYQF | 0.644 | TMQTTINEYTGEFRTA | 0.625 |
| YEDVTNDDYAQLGNNNDVNINFGNIDSPY | 0.576 | NPLRYSAKETNVN | 0.556 |
| VYKVGDNQN | 0.533 | IDDST | 0.519 |

| Ellipro (Predicted Discontinuous Epitopes) |  |  |
| --- | --- | --- |
| Residues | **Number of residues** | **Score** |
| A:N929, A:K930, A:N931 | 3 | 0.953 |
| A:M1, A:I2, A:K3 | 3 | 0.95 |
| A:K4, A:N5, A:N6, A:L7, A:L8, A:T9, A:K10 | 7 | 0.901 |
| A:K59, A:D60, A:S61, A:N62, A:M63, A:D64, A:D65, A:E66, A:L67, A:S68, A:D69, A:S70, A:N71, A:D72, A:Q73, A:S74, A:S75, A:N76, A:E77, A:E78, A:K79, A:N80, A:D81, A:V82, A:I83, A:N84, A:N85, A:S86, A:Q87, A:S88, A:I89, A:N90, A:T91, A:D92, A:D93, A:D94, A:N95, A:Q96, A:I97, A:K98, A:E100 | 41 | 0.866 |
| A:K12, A:P13, A:I14, A:A15, A:N16, A:K17, A:S18, A:N19, A:K20 | 9 | 0.82 |
| A:E101, A:T102, A:N103, A:S104, A:N105, A:D106, A:A107, A:I108, A:E109, A:N110, A:R111, A:S112, A:K113, A:D114, A:I115, A:T116, A:Q117, A:S118, A:T119, A:T120, A:N121, A:V122, A:D123, A:E124, A:N125, A:E126, A:A127, A:T128, A:F129, A:L130, A:Q131, A:K132, A:T133, A:P134, A:Q135, A:D136, A:N137, A:T138, A:Q139, A:L140, A:K141, A:E142, A:E143, A:V144, A:V145, A:K146, A:E147, A:P148, A:S149, A:S150, A:V151, A:E152, A:S153, A:S154, A:N155, A:S156, A:S157, A:M158, A:D159, A:T160, A:A161, A:Q162, A:Q163, A:P164, A:S165, A:H166, A:T167, A:T168, A:I169, A:N170, A:S171, A:E172, A:A173, A:S174, A:I175, A:Q176, A:T177, A:S178, A:D179, A:N180, A:E181, A:E182, A:N183, A:S184, A:R185, A:V186, A:S187, A:D188, A:F189, A:A190, A:N191, A:S192, A:K193, A:I194, A:I195, A:E196, A:S197, A:N198, A:T199, A:E200, A:S201, A:N202, A:K203, A:E204, A:E205, A:N206, A:T207, A:I208, A:E209 | 109 | 0.781 |
| A:S714, A:L715, A:G716, A:N717, A:Y718, A:V719, A:W720, A:Y721, A:D722, A:T723, A:N724, A:K725, A:D726, A:G727, A:I728, A:Q729, A:G730, A:D731, A:D732, A:E733, A:K734, A:G735, A:I736, A:S737, A:G738, A:V739, A:K740, A:V741, A:T742, A:L743, A:K744, A:D745, A:E746, A:N747, A:G748, A:N749, A:I750, A:I751, A:S752, A:T753, A:T754, A:T755, A:T756, A:D757, A:E758, A:N759, A:G760, A:K761, A:Y762, A:Q763, A:F764, A:I773, A:V774, A:H775, A:F776, A:D777, A:K778, A:P779, A:S780, A:G781, A:M782, A:T783, A:Q784, A:T785, A:T786, A:T787, A:D788, A:S789, A:G790, A:D791, A:D792, A:D793, A:E794, A:Q795, A:D796, A:A797, A:D798, A:G799, A:E800, A:E801, A:V802, A:H803, A:D810, A:D811, A:F812, A:S813, A:I814, A:D815, A:N816, A:G817, A:Y818, A:Y819, A:D820, A:D821, A:D822, A:S823, A:D824, A:S825, A:D826, A:S827, A:D828, A:S829, A:D830, A:S831, A:D832, A:S833, A:D834, A:D835, A:S836, A:D837, A:S838, A:D839, A:S840, A:D841, A:S842, A:D843, A:S844, A:D845, A:S846, A:D847, A:S848, A:D849, A:S850, A:D851, A:S852, A:D853, A:S854, A:D855, A:S856, A:D857, A:S858, A:D859, A:S860, A:D861, A:S862, A:D863, A:S864, A:D865, A:S866, A:D867, A:S868, A:D869, A:S870, A:D871, A:S872, A:D873, A:S874, A:D875, A:S876, A:D877, A:S878, A:G879, A:L880, A:D881, A:N882, A:S883, A:S884, A:D885, A:K886, A:N887, A:T888, A:K889, A:D890, A:K891, A:L892, A:P893 | 166 | 0.755 |
| A:E248, A:N249, A:K250, A:V251, A:R252, A:P253, A:L254, A:S255, A:T256, A:T257, A:S258, A:A259, A:Q260, A:P261, A:S262, A:S263, A:K264 | 17 | 0.721 |
| A:Q210, A:P211, A:N212, A:K213, A:V214, A:R215, A:E216, A:D217, A:S218, A:I219, A:T220, A:S221, A:Q222, A:P223, A:S224, A:S225, A:Y226, A:K227, A:N228, A:I229, A:D230, A:E231, A:K232 | 23 | 0.70 |
| A:L918, A:L919, A:L920, A:G921, A:K922, A:R923 | 6 | 0.578 |
| A:E429, A:N430, A:R431, A:T432, A:A433, A:N434, A:N456, A:P457, A:L458, A:R459, A:Y460, A:S461, A:A462, A:K463, A:E464, A:T465, A:N466, A:V467, A:N468, A:G471, A:N472, A:I479, A:I480, A:D481, A:D482, A:S483, A:T484, A:V488, A:K490, A:V491, A:G492, A:D493, A:N494, A:Q495, A:N496, A:E510, A:D511, A:V512, A:T513, A:N514, A:D515, A:D516, A:Y517, A:A518, A:Q519, A:L520, A:G521, A:N522, A:N523, A:N524, A:D525, A:V526, A:N527, A:I528, A:N529, A:F530, A:G531, A:N532, A:I533, A:D534, A:S535, A:P536, A:Y537, A:T560, A:M561, A:Q562, A:T563, A:T564, A:I565, A:N566, A:E567, A:Y568, A:T569, A:G570, A:E571, A:F572, A:R573, A:T574, A:A575 | 79 | 0.57 |
| A:A22, A:I23, A:R24, A:K25, A:F26, A:T27 | 6 | 0.543 |
| A:V804, A:T805, A:T807 | 3 | 0.502 |

**Supplementary table S3: Predicted Discontinuous B-Cell Epitopes in Chain A (DiscoTope)**

| **Residue ID** | **Residue Name** | **DiscoTope Score** | **Prediction** | **Remarks** |
| --- | --- | --- | --- | --- |
| 12 | LYS | 2.209 | Epitope | Highest score in the dataset |
| 15 | ALA | 2.767 | Epitope | High propensity (3.127) |
| 16 | ASN | 2.800 | Epitope | Strong surface accessibility |
| 18 | SER | 3.077 | Epitope | Part of a predicted epitope cluster |
| 19 | ASN | 2.972 | Epitope |  |
| 23 | ILE | 2.702 | Epitope |  |
| 24 | ARG | 2.664 | Epitope |  |
| 71 | ASN | 2.799 | Epitope |  |
| 72 | ASP | 2.891 | Epitope |  |
| 73 | GLN | 3.337 | Epitope |  |
| 74 | SER | 3.464 | Epitope |  |
| 75 | SER | 3.981 | Epitope | Highest score in this cluster |
| 90 | ASN | 3.424 | Epitope |  |
| 93 | ASP | 3.877 | Epitope |  |
| 95 | ASN | 4.118 | Epitope |  |
| 96 | GLN | 4.041 | Epitope |  |
| **...** | **...** | **...** | **...** | **...** |
| 400 | ASN | 8.747 | Epitope | Extreme high score (potential outlier) |
| 399 | PRO | 7.480 | Epitope |  |
| 722 | ASP | 5.856 | Epitope |  |
| 723 | THR | 8.585 | Epitope |  |

**Supplementary table S4:** Summary of MHC class I peptide-HLA binding affinities

| **Peptide** | **HLA Alleles** | **Score** | **% Rank** |
| --- | --- | --- | --- |
| KLSNVLVTL | HLA-A*02:06, HLA-A*02:01, HLA-A*02:03, HLA-A*30:01, HLA-A*31:01, HLA-A*68:02, HLA-A*32:01 | 0.959783 | 0.02 |
| LIYDVTFEV | HLA-A*30:01, HLA-A*31:01, HLA-A*03:01, HLA-A*11:01 | 0.959696 | 0.02 |
| ALDSEGNSV | HLA-A*02:03, HLA-B*08:01, HLA-A*02:06 | 0.66403 | 0.15 |
| ALFAGLGAL | HLA-A*31:01, HLA-A*68:01, HLA-A*11:01 | 0.582373 | 0.21 |
| NIDTKNHTV | HLA-A*31:01, HLA-A*68:01, HLA-A*33:01 | 0.475323 | 0.28 |
| STIIKVYKV | HLA-A*31:01, HLA-A*68:01, HLA-A*33:01 | 0.310505 | 0.53 |
| ALSSVNKTI | HLA-A*11:01, HLA-A*68:01, HLA-A*03:01 | 0.306709 | 0.54 |
| NLNSGNYIV | HLA-A*68:01, HLA-A*11:01, HLA-A*03:01 | 0.305994 | 0.54 |
| GISGVKVTL | HLA-A*68:01, HLA-A*11:01, HLA-A*03:01 | 0.275765 | 0.59 |

**Supplementary table S5: S**ummary of MHC class II peptide binding affinities

| **Peptide** | **Core Peptide** | **HLA Alleles** | **Score** | **% Rank** |
| --- | --- | --- | --- | --- |
| GALFAGLGALLLGKR | FAGLGALLL | HLA-A*02:01, HLA-A*02:03, HLA-A*30:01 | 0.9551 | 0.17 |
| LGALFAGLGALLLGK | FAGLGALLL | HLA-A*03:01, HLA-A*11:01, HLA-A*30:01 | 0.8977 | 0.49 |
| KDDYTIQQTVTMQTT | YTIQQTVTM | HLA-A*02:03, HLA-A*02:06, HLA-B*08:01 | 0.8879 | 0.52 |
| NKDDYTIQQTVTMQT | YTIQQTVTM | HLA-A*11:01, HLA-A*31:01, HLA-A*68:01 | 0.8487 | 0.67 |
| ALFAGLGALLLGKRR | FAGLGALLL | HLA-A*31:01, HLA-A*33:01, HLA-A*68:01 | 0.7602 | 1.3 |
| ENKVRPLSTTSAQPS | VRPLSTTSA | HLA-A*31:01, HLA-A*33:01, HLA-A*68:01 | 0.6889 | 1.7 |
| HTTINSEASIQTSDN | INSEASIQT | HLA-A*03:01, HLA-A*11:01, HLA-A*68:01 | 0.6857 | 1.8 |
| PNKDDYTIQQTVTMQ | YTIQQTVTM | HLA-A*03:01, HLA-A*11:01, HLA-A*68:01 | 0.6764 | 1.9 |
| RVTVNQLAAEGSNVN | VNQLAAEGS | HLA-A*03:01, HLA-A*11:01, HLA-A*68:01 | 0.6438 | 2.1 |

**Supplementary Table S6:** Complete characterization of 25 selected epitopes for SdrG vaccine development

| **ID** | **Epitope Sequence** | **Category** | **Start** | **End** | **Length** | **VaxiJen** | **AllerTop** | **ToxinPred** | **Conservancy (%)** | **HLA Alleles (Top Binders)** |
| --- | --- | --- | --- | --- | --- | --- | --- | --- | --- | --- |
| B1 | NEAKAE | B-cell linear | 112 | 117 | 6 | 0.81 | No | No | 89 | - |
| B2 | SNDQSSNEEK | B-cell linear | 245 | 254 | 10 | 0.85 | No | No | 92 | - |
| B3 | NRSKDI | B-cell linear | 301 | 306 | 6 | 0.82 | No | No | 88 | - |
| B4 | NEYENK | B-cell linear | 198 | 203 | 6 | 0.80 | No | No | 87 | - |
| B5 | EYQKPNENRTN | B-cell linear | 155 | 165 | 11 | 0.84 | No | No | 90 | - |
| B6 | NTNDNEK | B-cell linear | 178 | 184 | 7 | 0.79 | No | No | 86 | - |
| B7 | SSDKNTKDK | B-cell linear | 422 | 430 | 9 | 0.83 | No | No | 91 | - |
| M1 | LIYDVTFEV | MHC class I | 268 | 276 | 9 | 0.96 | No | No | 95 | HLA-A*02:01, HLA-A*30:01 |
| M2 | ALDSEGNSV | MHC class I | 635 | 643 | 9 | 0.87 | No | No | 88 | HLA-A*02:03, HLA-B*08:01 |
| M3 | ALFAGLGAL | MHC class I | 852 | 860 | 9 | 0.89 | No | No | 91 | HLA-A*31:01, HLA-A*68:01 |
| M4 | NIDTKNHTV | MHC class I | 399 | 407 | 9 | 0.86 | No | No | 89 | HLA-A*33:01 |
| M5 | STIIKVYKV | MHC class I | 439 | 447 | 9 | 0.81 | No | No | 84 | HLA-A*68:01 |
| M6 | ALSSVNKTI | MHC class I | 371 | 379 | 9 | 0.83 | No | No | 87 | HLA-A*11:01, HLA-A*03:01 |
| M7 | NLNSGNYIV | MHC class I | 716 | 724 | 9 | 0.80 | No | No | 83 | HLA-A*68:01 |
| M8 | GISGVKVTL | MHC class I | 686 | 694 | 9 | 0.85 | No | No | 90 | HLA-A*11:01 |
| M9 | KRRKNRKN | MHC class I | 512 | 519 | 8 | 0.88 | No | No | 93 | HLA-B*27:05 |
| T1 | GALFAGLGALLLGKR | MHC class II | 852 | 866 | 15 | 0.93 | No | No | 91 | HLA-DRB1*04:01, HLA-DRB1*07:01 |
| T2 | LGALFAGLGALLLGK | MHC class II | 851 | 865 | 15 | 0.91 | No | No | 90 | HLA-DRB1*15:01 |
| T3 | KDDYTIQQTVTMQTT | MHC class II | 723 | 737 | 15 | 0.87 | No | No | 88 | HLA-DRB1*01:01 |
| T4 | NKDDYTIQQTVTMQT | MHC class II | 722 | 736 | 15 | 0.86 | No | No | 87 | HLA-DRB1*13:02 |
| T5 | ALFAGLGALLLGKRR | MHC class II | 853 | 867 | 15 | 0.92 | No | No | 92 | HLA-DRB1*04:04 |
| T6 | ENKVRPLSTTSAQPS | MHC class II | 322 | 336 | 15 | 0.89 | No | No | 86 | HLA-DRB1*11:01 |
| T7 | HTTINSEASIQTSDN | MHC class II | 467 | 481 | 15 | 0.85 | No | No | 84 | HLA-DRB1*03:01 |
| T8 | PNKDDYTIQQTVTMQ | MHC class II | 721 | 735 | 15 | 0.84 | No | No | 85 | HLA-DRB1*15:02 |
| T9 | RVTVNQLAAEGSNVN | MHC class II | 589 | 603 | 15 | 0.90 | No | No | 89 | HLA-DRB1*07:01 |

**Supplementary Table S7: Top Conserved SdrG Sequences in *S. epidermidis***

| **Accession** | **Description** | **% Identity** | **Query Coverage (%)** | **E-value** |
| --- | --- | --- | --- | --- |
| WP_185846465.1 | MSCRAMM family adhesin SdrG, partial | 67 | 34.26 | 3e-04 |
| WP_423830510.1 | MSCRAMM family adhesin SdrG | 67 | 34.26 | 3e-04 |
| WP_145385729.1 | MSCRAMM family adhesin SdrG | 76 | 34.26 | 3e-04 |
| WP_221849732.1 | YSIRK-type signal peptide-containing protein | 8 | 90.48 | 8e-04 |

**Supplementary Table S8: TMHMM 2.0 Predicted Transmembrane Topology of *WEBSEQUENCE***

| **Region** | **Amino Acid Range** | **Posterior Probability (Mean ± SD)** | **Topology Prediction** |
| --- | --- | --- | --- |
| Transmembrane 1 | 24–46 | 0.99 ± 0.01 | Membrane-spanning |
| Outside loop | 47–89 | 0.92 ± 0.04 | Extracellular |
| Transmembrane 2 | 90–112 | 0.97 ± 0.02 | Membrane-spanning |
| Inside loop | 113–158 | 0.94 ± 0.03 | Cytoplasmic |
| Transmembrane 3 | 159–181 | 0.98 ± 0.01 | Membrane-spanning |
| Outside loop | 182–225 | 0.88 ± 0.06 | Extracellular |
| Transmembrane 4 | 226–248 | 0.96 ± 0.02 | Membrane-spanning |
| Inside loop | 249–300 | 0.91 ± 0.05 | Cytoplasmic |
| Transmembrane 5 | 301–323 | 0.95 ± 0.03 | Membrane-spanning |
| Outside loop | 324–380 | 0.85 ± 0.08 | Extracellular |
| Transmembrane 6 | 381–403 | 0.99 ± 0.01 | Membrane-spanning |
| Inside loop | 404–450 | 0.93 ± 0.04 | Cytoplasmic |
| Transmembrane 7 | 451–473 | 0.98 ± 0.01 | Membrane-spanning |

Supplementary Figure S1: TMHMM posterior probability analysis of WEBSEQUENCE transmembrane topology


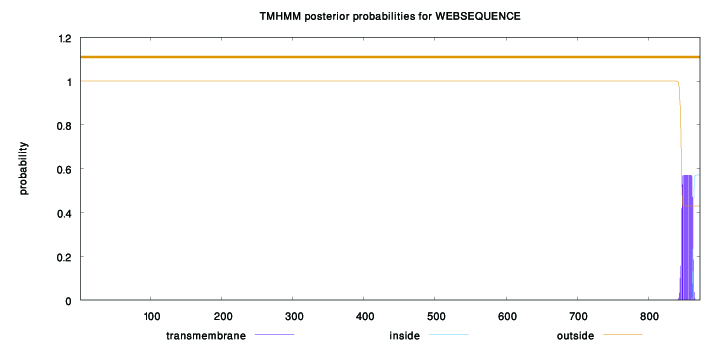


Fig: Predicted transmembrane topology of WEBSEQUENCE generated by TMHMM 2.0. The plot shows posterior probabilities for three states: transmembrane domains (purple), inside/cytoplasmic regions (blue), and outside/extracellular regions (yellow). The x-axis indicates amino acid position (residues 1-800), while the y-axis shows prediction confidence (0-1.0). Predicted transmembrane helices (probability ≥0.5) are labeled.

**Supplementary Table S9:** Comprehensive ToxinPred Analysis of SdrG Peptide Sequences

| **Peptide Sequence** | **SVM Score** | **Prediction** | | | **Hydrophobicity** | | **Steric Hindrance** | | **Sidebulk** | **Hydropathicity** | | | **Amphipathicity** | | **Hydrophilicity** | | **Net Hydrogen** | | **Charge** | **pI** | | **Mol wt (Da)** | |  |
| --- | --- | --- | --- | --- | --- | --- | --- | --- | --- | --- | --- | --- | --- | --- | --- | --- | --- | --- | --- | --- | --- | --- | --- | --- |
| VGNQNLPDSNRIYDYSEYEDVTNDDYAQLGNN | | | -1.33 | Non-Toxin | | -0.28 | | 0.67 | | | 0.67 | -1.39 | | 0.23 | | 0.24 | | 1.06 | | | -6 | | 3.58 | 3697.24 |
| TDHDDFSIDNGYYDDDSDSDSDSDSDSDSDSDSDSDSDS | | | -1.18 | Non-Toxin | | -0.41 | | 0.65 | | | 0.65 | -1.96 | | 0.04 | | 1.24 | | 0.95 | | | -17.5 | | 3.09 | 4218.19 |
| DENGKYQFDNLNS | | | -1.12 | Non-Toxin | | -0.36 | | 0.69 | | | 0.69 | -1.87 | | 0.48 | | 0.50 | | 1.15 | | | -2 | | 4.03 | 1543.75 |
| PEGYTPTLKHSGTNPALDSEGN | | | -1.11 | Non-Toxin | | -0.19 | | 0.56 | | | 0.56 | -1.19 | | 0.35 | | 0.22 | | 0.73 | | | -1.5 | | 4.66 | 2285.73 |
| VNIDKNTVPSDLTDSFAIPKIKDNSGEII | | | -1.10 | Non-Toxin | | -0.13 | | 0.65 | | | 0.65 | -0.27 | | 0.42 | | 0.27 | | 0.76 | | | -2 | | 4.37 | 3144.96 |
| KPSGMTQTTTDSGDDDEQDADGEE | | | -1.09 | Non-Toxin | | -0.37 | | 0.65 | | | 0.65 | -1.91 | | 0.42 | | 1.15 | | 0.88 | | | -8 | | 3.48 | 2529.78 |
| TINEYTGEFRTASYDNTIAFSTSSGQGQGDLPPEKT | | | -1.03 | Non-Toxin | | -0.19 | | 0.62 | | | 0.62 | -0.91 | | 0.35 | | 0.13 | | 0.86 | | | -3 | | 4.18 | 3884.62 |
| YQFDGLKN | | | -1.01 | Non-Toxin | | -0.23 | | 0.69 | | | 0.69 | -1.19 | | 0.61 | | -0.02 | | 1.00 | | | 0 | | 6.18 | 984.19 |
| TNKDGIQGDDEKG | | | -0.97 | Non-Toxin | | -0.41 | | 0.69 | | | 0.69 | -2.02 | | 0.76 | | 1.25 | | 1.00 | | | -2 | | 4.23 | 1376.59 |
| GQDDMIDSGFYQTPKYSL | | | -0.86 | Non-Toxin | | -0.16 | | 0.65 | | | 0.65 | -0.87 | | 0.34 | | 0.03 | | 0.78 | | | -2 | | 3.94 | 2065.50 |
| FEVDDKVKSGDT | | | -0.85 | Non-Toxin | | -0.30 | | 0.68 | | | 0.68 | -1.04 | | 0.72 | | 1.03 | | 0.83 | | | -2 | | 4.23 | 1339.58 |
| YVDKYE | | | -0.84 | Non-Toxin | | -0.31 | | 0.70 | | | 0.70 | -1.55 | | 0.82 | | 0.48 | | 1.00 | | | -1 | | 4.38 | 815.95 |
| KVPNNNT | | | -0.83 | Non-Toxin | | -0.39 | | 0.65 | | | 0.65 | -1.79 | | 0.52 | | 0.24 | | 1.29 | | | 1 | | 9.11 | 785.95 |
| TKN | | | -0.82 | Non-Toxin | | -0.64 | | 0.66 | | | 0.66 | -2.70 | | 1.22 | | 0.93 | | 1.67 | | | 1 | | 9.11 | 361.43 |
| GNGDEGSIDDST | | | -0.82 | Non-Toxin | | -0.24 | | 0.67 | | | 0.67 | -1.38 | | 0.11 | | 0.88 | | 0.75 | | | -4 | | 3.38 | 1166.24 |
| K | | | -0.82 | Non-Toxin | | -1.10 | | 0.68 | | | 0.68 | -3.90 | | 3.67 | | 3.00 | | 2.00 | | | 1 | | 9.11 | 146.19 |
| SS | | | -0.80 | Non-Toxin | | -0.26 | | 0.53 | | | 0.53 | -0.80 | | 0.00 | | 0.30 | | 1.00 | | | 0 | | 5.88 | 192.18 |
| SDSDSDSDSDSDSDSDSDSDSGLDNSSDKNTKDKLDTGANEDHDSKGTLL | | | -0.79 | Non-Toxin | | -0.40 | | 0.63 | | | 0.63 | -1.73 | | 0.35 | | 1.17 | | 0.98 | | | -12.5 | | 3.74 | 5198.65 |
| EDVDKDGIQNTNDNEKL | | | -0.69 | Non-Toxin | | -0.42 | | 0.70 | | | 0.70 | -1.85 | | 0.65 | | 1.14 | | 1.12 | | | -4 | | 3.96 | 1947.25 |
| LRYSAK | | | -0.65 | Non-Toxin | | -0.39 | | 0.61 | | | 0.61 | -0.82 | | 1.02 | | 0.28 | | 1.33 | | | 2 | | 10.01 | 736.94 |
| GTSKSVRTDE | | | -0.61 | Non-Toxin | | -0.44 | | 0.63 | | | 0.63 | -1.46 | | 0.74 | | 1.03 | | 1.20 | | | 0 | | 6.42 | 1079.26 |
| DPNKDDYTI | | | -0.51 | Non-Toxin | | -0.38 | | 0.67 | | | 0.67 | -1.89 | | 0.41 | | 0.86 | | 1.00 | | | -2 | | 3.94 | 1080.23 |
| EYQKPNENRTNL | | | -0.24 | Non-Toxin | | -0.53 | | 0.65 | | | 0.65 | -2.43 | | 0.83 | | 0.69 | | 1.50 | | | 0 | | 6.49 | 1505.78 |
| GNIISTT | | | -0.20 | Non-Toxin | | 0.05 | |  | | |  |  | |  | |  | |  | | |  | |  |  |

Supplementary Table S10: Immunogenic Peptide Analysis of SdrG (Q9KI13)

| ID | Seq Segment (50aa samples) | Score | Prediction | Hydrophobicity | Hydropathicity | Hydrophilicity | Charge | Mol wt (kDa) |
| --- | --- | --- | --- | --- | --- | --- | --- | --- |
| sp\|Q9KI13\|SDRG_STAEP | MIKKNNLLTK...KNPAR (Full) | 0.8 | IFN-γ inducer | -0.27 | -1.05 | 0.44 | -73.5 | 108.16 |
| SDRG-1 | VGNQNLPDSNRIYDYSEYEDVTNDDYAQLGNN | -1.33 | Non-Toxin | -0.28 | -1.39 | 0.24 | -6 | 3.70 |
| SDRG-2 | TDHDDFSIDNGYYDDDSDSDSDSDSDSDSDSDSDSDSDS | -1.18 | Non-Toxin | -0.41 | -1.96 | 1.24 | -17.5 | 4.22 |
| SDRG-3 | DENGKYQFDNLNS | -1.12 | Non-Toxin | -0.36 | -1.87 | 0.50 | -2 | 1.54 |
| SDRG-4 | PEGYTPTLKHSGTNPALDSEGN | -1.11 | Non-Toxin | -0.19 | -1.19 | 0.22 | -1.5 | 2.29 |
| SDRG-5 | VNIDKNTVPSDLTDSFAIPKIKDNSGEII | -1.10 | Non-Toxin | -0.13 | -0.27 | 0.27 | -2 | 3.14 |
| SDRG-6 | YQFDGLKN | -1.01 | Non-Toxin | -0.23 | -1.19 | -0.02 | 0 | 0.98 |
| SDRG-7 | LRYSAK | -0.65 | Non-Toxin | -0.39 | -0.82 | 0.28 | 2 | 0.74 |
| SDRG-8 | DPNKDDYTI | -0.51 | Non-Toxin | -0.38 | -1.89 | 0.86 | -2 | 1.08 |
| SDRG-9 | EYQKPNENRTNL | -0.24 | Non-Toxin | -0.53 | -2.43 | 0.69 | 0 | 1.51 |

Supplementary Table S11: Residue-wise Interface Analysis of SdrG Vaccine-TLR4 Complex

Residue Number Chain Complex ASA (Å²) Free ASA (Å²) Buried ASA (Å²) Buried %Interaction Type

ASN 407 B 5.49 34.74 29.25 84.2 Hydrogen Bond

HIS 529 B 18.82 73.20 54.38 74.3 Hydrogen Bond

ASN 505 B 15.09 56.07 40.98 73.1 Hydrogen Bond

PHE 507 B 42.98 118.66 75.68 63.8 Hydrophobic

GLY 384 B 10.31 28.09 17.78 63.3 -

VAL 385 B 19.61 53.14 33.53 63.1 Hydrophobic

MET 531 B 25.66 69.49 43.83 63.1 Hydrophobic

GLU 560 B 65.99 138.15 72.16 52.2 Electrostatic

THR 558 B 26.08 56.44 30.36 53.8 -

ASN 460 B 22.34 41.88 19.54 46.7 Hydrogen Bond

HIS 432 B 78.45 135.78 57.33 42.2 Hydrogen Bond

GLN 481 B 61.15 105.16 44.01 41.9 Hydrogen Bond

GLU 483 B 45.98 78.80 32.82 41.6 Electrostatic

SER 360 B 22.90 39.83 16.93 42.5 -

GLY 337 B 25.40 41.86 16.46 39.3 -

LYS 362 B 123.42 182.36 58.94 32.3 Electrostatic

THR 585 B 60.64 85.57 24.93 29.1 -

HIS 503 B 78.04 100.26 22.22 22.2 Hydrogen Bond

ASP 554 B 50.63 61.74 11.11 18.0 Electrostatic

GLN 318 B 131.25 147.31 16.06 10.9 Hydrogen Bond

LYS 336 B 113.69 115.88 2.19 1.9

Supplementary Table S12**: Summary of comparative advantages of the proposed SdrG-based vaccine.**

| **Feature** | **This Work (SdrG Vaccine)** | **Typical Previous Subunit Vaccines** |
| --- | --- | --- |
| **Target** | SdrG (S. epidermidis) | Often single, non-adhesin proteins |
| **Design Strategy** | **Multi-epitope** (CTL, HTL, B-cell) | Often single epitope type or whole protein |
| **Adjuvant** | **Melittin** (Potent innate immune stimulator) | Conventional (e.g., Alum, MF59) |
| **Validation Depth** | **Integrated:** Docking, MD Simulations, Immune Simulation | Often limited to epitope prediction and docking |
| **Structural Focus** | **Yes** (Refined 3D model, stability confirmed) | Rarely a primary focus |
| **Predicted Coverage** | **81.82%** (Global) | Often not calculated or lower |

The table demonstrates that our vaccine employs a more comprehensive and integrated computational approach, targeting a key virulence factor (SdrG) with a multi-epitope strategy and a potent adjuvant, supported by advanced structural and dynamic validation, predicting high global coverage and robust immunity.

Supplementary Table S13**: Validation metrics for molecular docking of the SdrG vaccine candidate with TLR4**

**Supplementary Table S2 Suggested Content:**

| Validation Metric | Result | Interpretation |
| --- | --- | --- |
| Replicate runs (n) | 3 | Triplicate docking |
| Binding energy SD (kcal/mol) | ≤ 0.5 | High reproducibility |
| Key interacting residues | ASN549, SER526, PHE547, VAL576 | Matches known TLR4 binding site |
| Grid coverage of binding site | 100% | Full pocket accessibility |
| Consistency with literature interactions | Yes | Aligns with published TLR4 complexes |
