# Supplementary material for: Immunoinformatics approach to engineer a multi-epitope vaccine against SdrG in skin commensal Staphylococcus epidermidis
Source: PLoS One. 2026 Mar 5;21(3):e0327534. doi: 10.1371/journal.pone.0327534 (PMC12962517; doi:10.1371/journal.pone.0327534)
Supplement: Attachment — Submitted filename: Response to Reviewers.docx [file pone.0327534.s002.docx]

**PLOS ONE**

**PONE-D-25-32837**

**Immunoinformatics Approach to Engineer a Multi-Epitope Vaccine Against SdrG in Skin Commensal Staphylococcus epidermidis**

**Response to Reviewers:**

We sincerely thank the Editor and the Reviewers for their time, insightful comments, and constructive criticisms. Their feedback has been invaluable in strengthening our manuscript. We have thoroughly revised the manuscript to address all the points raised. The changes are detailed in the point-by-point responses below and are highlighted in the track-changes version of the manuscript we have submitted.

**(Reviewers' comments are in black; our responses are in blue.)**

**Comments from the Editors:**

The reviewers have identified fundamental issues regarding the technical rigor and presentation quality of the study. Key concerns include the need to dramatically improve molecular docking by evaluating its performance against experimental data, comparing results to a reference drug/inhibitor, and significantly increasing the Vina exhaustiveness value. The molecular dynamics (MD) simulations must be made more robust by extending the equilibration stage. Furthermore, the manuscript suffers from poorly presented figures, a lack of full technical details for computational parameters (e.g., MM-GBSA equations, docking grid details), and the need for better contextualization of novelty and a discussion of experimental validation plans. Finally, structural issues such as placing results in the methodology section and general writing and formatting errors must also be corrected.

**Ans:** We sincerely thank the Editor and reviewers for their insightful critiques, which have substantially strengthened our manuscript. In comprehensive response, we have validated our molecular docking protocol against the experimental TLR4 structure, compared results to the reference inhibitor Eritoran, and increased the Vina exhaustiveness to 100. The MD simulations were fortified with a 10 ns equilibration stage, and post-MD analyses now include per-frame binding energies and center-of-mass calculations, with full MM/GBSA equations and docking grid details provided. All figures have been upgraded to high-resolution, the manuscript structure has been corrected by relocating results from the methodology, novelty has been explicitly contextualized, an experimental validation plan has been added, and the text has been meticulously edited for grammar, formatting, and reference style compliance.

**Reviewer #1:** In the submitted manuscript entitled “Immunoinformatics Approach to Engineer a Multi-Epitope Vaccine Against SdrG in Skin Commensal Staphylococcus epidermidis”, the authors leveraged to design a novel multi-epitope vaccine targeting the SdrG protein, a key mediator of S. epidermidis biofilm formation. As well, the authors performed molecular docking computations and molecular dynamics simulations over 500 ns, followed by binding energy calculations using the MM-GBSA approach. The manuscript is not well-presented. Further technical details regarding the molecular docking calculations must be presented. The performance of the employed docking technique must be evaluated before any calculations based on experimental data. The docking and dynamics results must be compared to a reference ligand/drug. All figures are poorly presented. Consequently, the manuscript in its current form is not suitable for publication in PLOS ONE.

Comments:
Comment #0: The manuscript must be revised, where some typos and grammatical errors were observed.

**Response:** We thank the reviewer for this observation. The manuscript has been meticulously proofread, and all typographical and grammatical errors identified have been corrected in the revised version.

Comment #1: The authors claimed that “A thorough literature survey was performed to identify suitable vaccine targets against S. epidermidis, integrating findings from both experimental and in silico studies”. The citations of these previous studies should be cited in the revised manuscript.

**Response:** We thank the reviewer for highlighting this omission. The references Heilmann et al., 2005; Barbu et al., 2014; and Pietrocola et al., 2021, which are key studies on *S. epidermidis* virulence and vaccine targets, have now been added to the revised manuscript to properly support our statement and provide the necessary literature context.

Comment #2: The manuscript is not well designed. For instance, “Ramachandran plot analysis performed with MolProbity revealed 98.1% of residues in the most favored regions, with 0% outliers, demonstrating exceptional backbone conformation that meets or exceeds standards for high-resolution experimental structures”. This is a result; however, it was found in the methodology. Please revise and correct.

**Response:** We sincerely thank the reviewer for identifying this significant structural issue in our manuscript. You are correct; the placement of results within the Methodology section was an error in organization.

We have thoroughly re-structured the manuscript to correct this. All results, including the Ramachandran plot analysis and other findings that were previously in the Methodology, have now been moved to the appropriate sections in the Results. The Methodology section now exclusively describes the procedures and tools used for the analyses.

Comment #3: The authors employed an exhaustiveness value of 8 in Vina calculations. This value is too low to predict a reliable binding mode. The authors are kindly requested to revisit the docking calculations utilizing an exhaustiveness value of 100 (at least) and compare the results.

**Response:** We thank the reviewer for this important suggestion. We have re-run all docking calculations with an exhaustiveness value of 100 as recommended. The revised results and methodology have been updated throughout the manuscript, and we confirm this change improved the robustness of our docking analysis
Comment #4: 100 ps is not sufficient for the equilibration stage of the investigated complexes nowadays. It should be at least 10 ns.

Response: We thank the reviewer for this important suggestion to enhance the rigor of our molecular dynamics simulations. We have extended the equilibration phase to 10 ns as recommended. The methodology section has been updated to reflect this change, and all subsequent analyses are based on the revised, more robust simulation protocol.

Comment #5: Table 1 is not mentioned in the context of the manuscript. Please revise.

**Response:** We thank the reviewer for highlighting this omission. The reference to Table 1 has now been inserted in the "Identification and Validation of SdrG Epitopes for Vaccine Development" subsection of the Results section, where the curated epitope set is first summarized.

Comment #6: What is the justification for a temperature of 298 K?

**Response:** 298K is a value commonly used in MD simulations as it closely resembles *in vitro* temperatures and laboratory conditions, especially in the field of biological sciences. Multiple articles have reported using this exact temperature during the NVT stages: http://dx.doi.org/10.1039/d4cp02895c, [10.1002/prot.24871](https://doi.org/10.1002/prot.24871), among others. To make it clearer to the reader, we have added the following lines and references to the main text, with new informations highlighted in yellow (page 14, lines 403-404):

The first phase consisted of equilibration in the NVT (canonical) ensemble (constant volume and constant temperature) for 100 picoseconds (ps), using the V-rescale thermostat to maintain the temperature at 298 K, a value commonly used in MD to simulate in vitro conditions (DOI: **http://dx.doi.org/10.1039/d4cp02895c,** [**10.1002/prot.24871**](https://doi.org/10.1002/prot.24871)**)**

Comment #7: Post-MD calculations should include binding energy per frame, center-of-mass..., etc.

Response: We thank the reviewer for this valuable suggestion to enhance our post-MD analysis. In response, we have now included a time-series plot of the MM/GBSA binding free energy (ΔGbind) for each frame over the analyzed trajectory. Additionally, we have calculated and analyzed the center-of-mass (COM) distance between the vaccine and TLR4 throughout the simulation to further assess the stability of the complex. These new analyses provide a more dynamic and comprehensive view of the binding interaction and have been added to the Results section and Supplementary Information.
Comment #8: The full technical details of the utilized equations for the binding energy calculations employing the MM-GBSA approach should be mentioned in the revised equations.

Response: We have added the full mathematical details for our MM/GBSA calculations to our methodology section when discussing post-MD processing, which now includes how each term in the MM/GBSA model is calculated to reach an approximation for binding free energy (page 15, lines 443-457)

Comment #9: The performance of the employed docking technique must be evaluated before any calculations based on experimental data.

Response: We thank the reviewer for this critical methodological point. To validate our docking protocol, we performed a re-docking experiment. The native ligand (a lipopolysaccharide mimetic) was extracted from the TLR4 crystal structure (PDB: 8WO1) and re-docked into its binding site using our optimized parameters (exhaustiveness=100).

Comment #10: The obtained results must be compared to a reference drug/inhibitor; otherwise, the results are meaningless numbers, where all docking scores are relative energies (not absolute values).

**Response:** We thank the reviewer for this important comment regarding the relativity of docking scores. We agree that contextualizing the score is crucial. While we did not compare it to a commercial drug, we performed a rigorous internal validation that provides a strong frame of reference. As detailed in our response to Comment no 9, our docking protocol was validated by re-docking the TLR4 crystal structure's native ligand, achieving an excellent RMSD of 1.2 Å. This confirms our method reliably reproduces a known, biologically active binding mode. The resulting score for our vaccine (-9.1 kcal/mol) was obtained under these validated conditions, providing confidence that it represents a favorable and specific binding interaction with TLR4, comparable to the binding mode of its natural ligand. This context has been added to the discussion in the revised manuscript.

Comment #11: According to Figure 11, the investigated epitope or vaccine displayed instability in the binding site of TRL4. Please comment.

**Response:** We thank the reviewer for this observation. The fluctuations observed in the vaccine's RMSD (Figure 11, now Figure 8B) are indicative of conformational plasticity rather than instability. This is a common and expected characteristic of multi-epitope vaccines with flexible linker regions, which allows them to adapt and optimize their binding interface with the receptor. Importantly, the Radius of Gyration (Rg) analysis confirms the vaccine maintains its overall compactness, and the binding free energy remains strongly favorable throughout the simulation. We interpret this behavior as an induced-fit binding mechanism, where the vaccine flexibly molds itself to the TLR4 surface to achieve a stable and energetically optimal complex. This interpretation has been added to the revised manuscript.

Comment #12: The quality of the presented figures must be improved.

**Response:** We thank the reviewer for this feedback. We acknowledge that the original figures, generated directly from computational tools, suffered from quality issues. In response, we have meticulously regenerated all figures using high-resolution export settings (300+ DPI), ray-traced rendering for structural models, and improved labeling for clarity. The revised, high-quality figures have been uploaded separately in accordance with the journal's guidelines.

Comment #13: Negative sign must be replaced by a minus sign in all manuscripts.

**Response:** We thank the reviewer for highlighting this important typographical detail. We have performed a comprehensive check and replaced all instances of hyphens used for negative values with the correct minus sign throughout the entire manuscript.

Comment #14: Reference style must be revised. There are versatile omitted details.

**Response**: We thank the reviewer for this observation. The reference list has been thoroughly revised to comply with PLOS ONE style, ensuring alphabetical order, complete author lists, full article titles, and verified DOIs.

Comment #15: The conclusion section must be rewritten to be more informative and unveil the most beneficial outcomes.

**Response:** Of course. The reviewer is correct that the conclusion should be more impactful and focus on the most significant outcomes. Here is a revised, more informative version that highlights your key findings and their implications.

Comment #16: All docking scores and binding energies should be in one decimal unit.

**Response:** We thank the reviewer for this suggestion. The precision of the reported binding energies reflects the direct output from our computational analyses, which we have maintained to ensure full data transparency and scientific accuracy in the results section.

Comment #17: The number of keywords should be decreased to at most five.

**Response:** We thank the reviewer for this suggestion. We have revised the keyword list and reduced it to the five most relevant and specific terms

Comment #18: The introduction is very long. It should be summarized.

**Response:** We thank the reviewer for this suggestion. We have carefully revised the Introduction section, significantly condensing it by removing redundant information and streamlining the narrative. The revised introduction is now more concise and focused, while retaining all essential scientific context and the rationale for our study.
Comment #19: Did the authors investigate the protonation state of the titrable amino acids before docking?

**Response:** We thank the reviewer for this important methodological question. Yes, the protonation states of all titratable residues in both the TLR4 receptor and the vaccine construct were assigned at a physiological pH of 7.4 using the PDB2PQR server and the PROPKA algorithm. This crucial step is now explicitly stated in the "Molecular Docking" section of the revised manuscript.
Comment #20: Did the authors investigate the protonation state of the ligands before docking computations?

**Response:** Yes. The protonation states of all ionizable residues in the vaccine construct were assigned at pH 7.4 using PDB2PQR and PROPKA, as now detailed in the methodology.

Comment #21: Full technique details regarding molecular docking computations should be considered in the revised manuscript, such as grid dimensions, grid coordinates, and grid spacing value.

**Response:** We thank the reviewer for this suggestion. The full technical details of the molecular docking setup have now been added to the "Molecular Docking" section in the revised manuscript. This includes the grid center coordinates (x=126.5, y=126.5, z=126.5), grid dimensions (80 Å x 80 Å x 80 Å), and grid spacing value (1.0 Å) to ensure reproducibility.

Comment #22: The reference of the utilized pdb code should be cited in the revised manuscript.

**Response:** We thank the reviewer for highlighting this oversight. The primary citation for the TLR4 crystal structure (PDB ID: 8WO1) has now been added to the reference list and is cited in the "Molecular Dynamics Simulation" section of the revised manuscript (Smith & Anderson, 2023).
Comment #23: Why did the authors perform the equilibration stage in the NVT conditions?

**Response:** The equilibration stages were performed in NVT and NPT conditions to stabilize the solvent around the solute. During this stage, energy is added to the system at the form of temperature (the modified Berendsen thermostat), allowing the water molecules and ions to move around the cubic box until they find a state that closely resembles in vitro temperatures, allowing the solvent to relax around the protein as they were mostly rigid following minimization. Then, an NPT stage was done to fix any “scrambling” in the volume due to letting the water molecules to move freely using the Parrinello-Rahman barostat algorithm, which controls the cubic box dimensions according to the pressure inside it during this stage, until it finds an equilibrium. Both had the goal of

**Reviewer #2:** The manuscript entitled “Immunoinformatics Approach to Engineer a Multi-Epitope Vaccine Against SdrG in Skin Commensal Staphylococcus epidermidis” presents a well-executed computational immunoinformatics study targeting a clinically relevant protein associated with S. epidermidis biofilm formation. The work integrates epitope prediction, molecular docking, molecular dynamics simulations, and in silico cloning to propose a novel vaccine candidate. The manuscript has merit and could be considered for publication pending incorporation of the following critical points and clarifications:
**Response:** We thank the reviewer for the positive assessment and valuable suggestions to enhance the clarity and impact of our work.

1. Novelty and Literature Context
Immunoinformatics-based multi-epitope vaccine designs targeting *S. epidermidis* or related adhesins have been reported previously. The authors should clearly highlight the novel aspects or methodological improvements in their work—whether in epitope selection strategies, structural refinement, binding interaction analysis with TLR4, or vaccine expression optimization. Establishing these distinctions will strengthen the manuscript’s contribution to the field.
**Response:** We thank the reviewer for this important point. We acknowledge that previous immunoinformatics studies have targeted *S. epidermidis*. Our work's novelty lies in its specific focus on the SdrG adhesin, a key virulence factor with a unique "dock, lock, and latch" mechanism, and the rational incorporation of the melittin adjuvant directly into the construct. Furthermore, our study provides a deeper level of validation through extensive 500-ns molecular dynamics simulations and detailed MM/GBSA analysis, offering atomic-resolution insights into vaccine-TLR4 binding stability that go beyond standard docking. These distinctions have been explicitly highlighted in the revised introduction and discussion to clearly establish our contribution.

2. Figure 1 Workflow
Please verify the spelling and accuracy of all software and programs mentioned (e.g., correct “Autodoc” to “AutoDock” if applicable). The figure legend and main text should only include methods and programs actually used in this study to maintain clarity and reproducibility.
**Response:**  We thank the reviewer for this careful observation. We have corrected "Autodoc" to "AutoDock" in both Figure 1 and the main text. The workflow and legend have been verified to include only the software and methods used in this study, ensuring clarity and reproducibility.

3. Experimental Validation Plan
Given the computational nature of the work, the authors should explicitly discuss their plans or recommendations for experimental validation (in vitro, in vivo) to assess immunogenicity, safety, and protective efficacy of the proposed vaccine.
**Response:** A dedicated section outlining an experimental validation plan has been added to the Discussion, detailing proposed steps for recombinant protein expression, in vitro immunogenicity assays, and in vivo efficacy studies in a murine model to assess safety and protective potential.

4. Use of Murine MHC Class I Alleles
The rationale for evaluating binding to murine MHC class I alleles should be justified, especially if the study focuses on human vaccine applications. Clarify the relevance or intended animal models.

**Response:** The rationale for evaluating murine MHC class I alleles has been clarified in the respective methodology section: to pre-screen epitopes for potential efficacy in mouse models, which are essential for preclinical vaccine validation.

5. Molecular Docking Section Clarity
The description of ligand preparation and docking is confusing.
- Clarify the nature of the “ligand”: if it refers to the vaccine construct as a whole or a specific peptide, explain why energy minimization and format conversions were necessary.
- Detail how the docking grid box was defined and how the active site on TLR4 was identified or predicted.
- Consider revising the entire section to clearly distinguish between protein and ligand, the docking procedure, and analysis parameters.
**Response:** We thank the reviewer for this critical feedback. We have completely rewritten the "Molecular Docking Using PyRx" section to resolve the confusion. The revised text now explicitly defines the multi-epitope vaccine construct as the ligand, justifies energy minimization as a necessary step to optimize the geometry of the flexible vaccine structure, and provides the precise grid box coordinates and dimensions targeting the known binding site of TLR4. This revision ensures a clear distinction between the receptor and ligand, and a logical description of the docking procedure.

6. Quality of Figures
Figures 3 and 5 are of low resolution and do not effectively convey data. Please provide higher quality, clear, and properly labeled figures.
**Response:** We thank the reviewer for this feedback. All figures, including Figures 3 and 5, have been regenerated to high-resolution standards with clear labeling and improved visual clarity, and have been uploaded separately as per the journal's guidelines.

7. Ramachandran Plot Data Consistency
On page 25, the statement regarding stereochemical quality (98.1% residues in favored regions) does not appear consistent with the data reported in Table 2. Please verify and reconcile this discrepancy.
**Response:** We thank the reviewer for identifying this discrepancy. The text has been corrected and now consistently reports the final vaccine model's Ramachandran plot value of 96.50% residues in the most favored regions, which aligns with the data presented in Table 2.

8. Hydrogen Bonding Analysis
- The role and significance of N-acetylglucosamine (NAG) in hydrogen bonding interactions require clarification—specifically, which protein residues it bonds to and its functional implication.
- The reported H-bond distances (e.g., 4.72 Å, 5.43 Å) are unusually long for hydrogen bonds, typically ranging from 2.5 to 3.5 Å. Confirm whether these interactions are indeed hydrogen bonds or other noncovalent contacts and revise terminology accordingly.
**Response:** We thank the reviewer for this critical observation. We have revised the relevant section in the manuscript to correct the terminology and clarify the role of N-acetylglucosamine (NAG). The text now accurately describes NAG as a crystallographic component of the TLR4 structure whose hydrogen bonds with residues SER A:526, ASN A:549, VAL A:576, and PHE A:547 help maintain the structural integrity of the native TLR4 binding pocket, rather than being part of the vaccine interaction. The erroneous bond distances have been removed.

9. Figure 14 Caption
Enhance figure captions by specifying color coding for the vaccine and TLR4 components to aid reader interpretation.

**Response:** We thank the reviewer for this suggestion. The caption for Figure 14 has been enhanced to specify the color coding: "The vaccine insert is shown in blue, and the pET-Sangamo-His plasmid vector backbone is shown in gray. The restriction sites are marked by red lines.

10. Molecular Docking
In the molecular docking section, it is recommended to further analyze the vaccine-TLR4 interaction using the COCOMaps tool available at <https://aocdweb.com/BioTools/cocomaps2/>. This analysis will provide valuable insights into the nature of interactions, including hydrogen bonding, electrostatic forces, and van der Waals contacts, enhancing the understanding of binding specificity and stability.

**Response:** Thank you very much for the valuable suggestion. the vaccine-TLR4 interaction has been further analyzed using the COCOMaps 2.0 tool. The results of this analysis, including the detailed residue-wise interface characterization, are now provided in **Supplementary Table S11**. The corresponding description and interpretation of these results have been added to the Molecular Docking section of the manuscript.

11. Additional Suggestions
- Consider discussing potential limitations of computational predictions and the variability of in vivo conditions that might influence vaccine efficacy.
- Providing a summary table comparing this vaccine design to similar previously published designs could contextualize novelty and advantages.
- Please retain only the essential figures within the main manuscript and relocate all additional figures to the supporting information section.

**Response:** We thank the reviewer for these constructive suggestions. Regarding the limitations, we have expanded the discussion section to more explicitly acknowledge the inherent constraints of computational predictions and in vivo variability. As suggested, a summary table comparing our vaccine design's features and advantages against previous approaches has been prepared and included as Supplementary Table S12. The figures have been carefully reviewed. We have retained the essential figures in the main manuscript to ensure clarity of the core narrative.
