# Supplementary material for: Immunoinformatics approach to engineer a multi-epitope vaccine against SdrG in skin commensal Staphylococcus epidermidis
Source: PLoS One. 2026 Mar 5;21(3):e0327534. doi: 10.1371/journal.pone.0327534 (PMC12962517; doi:10.1371/journal.pone.0327534)
Supplement: Peer review history

# Peer review: Decision Letter 0

**DOI:** 10.1371/journal.pone.0327534.r001

**Authors:** Syed Hani Abidi

8 Oct 2025

Dear Dr. Akter,

Thank you for submitting your manuscript to PLOS ONE. After careful consideration, we feel that it has merit but does not fully meet PLOS ONE’s publication criteria as it currently stands. Therefore, we invite you to submit a revised version of the manuscript that addresses the points raised during the review process.

Please submit your revised manuscript by Nov 22 2025 11:59PM. If you will need more time than this to complete your revisions, please reply to this message or contact the journal office at plosone@plos.org . When you're ready to submit your revision, log on to https://www.editorialmanager.com/pone/ and select the 'Submissions Needing Revision' folder to locate your manuscript file.

- A rebuttal letter that responds to each point raised by the academic editor and reviewer(s). You should upload this letter as a separate file labeled 'Response to Reviewers'.
- A marked-up copy of your manuscript that highlights changes made to the original version. You should upload this as a separate file labeled 'Revised Manuscript with Track Changes'.
- An unmarked version of your revised paper without tracked changes. You should upload this as a separate file labeled 'Manuscript'.

If you would like to make changes to your financial disclosure, please include your updated statement in your cover letter. Guidelines for resubmitting your figure files are available below the reviewer comments at the end of this letter.

If applicable, we recommend that you deposit your laboratory protocols in protocols.io to enhance the reproducibility of your results. Protocols.io assigns your protocol its own identifier (DOI) so that it can be cited independently in the future. For instructions see: https://journals.plos.org/plosone/s/submission-guidelines#loc-laboratory-protocols . Additionally, PLOS ONE offers an option for publishing peer-reviewed Lab Protocol articles, which describe protocols hosted on protocols.io. Read more information on sharing protocols at https://plos.org/protocols?utm_medium=editorial-email&utm_source=authorletters&utm_campaign=protocols .

We look forward to receiving your revised manuscript.

Kind regards,

Syed Hani Abidi

Academic Editor

PLOS ONE

Journal Requirements:

When submitting your revision, we need you to address these additional requirements.

1.Please ensure that your manuscript meets PLOS ONE's style requirements, including those for file naming. The PLOS ONE style templates can be found at

https://journals.plos.org/plosone/s/file?id=wjVg/PLOSOne_formatting_sample_main_body.pdf and

https://journals.plos.org/plosone/s/file?id=ba62/PLOSOne_formatting_sample_title_authors_affiliations.pdf

2. Please note that PLOS One has specific guidelines on code sharing for submissions in which author-generated code underpins the findings in the manuscript. In these cases, we expect all author-generated code to be made available without restrictions upon publication of the work. Please review our guidelines at https://journals.plos.org/plosone/s/materials-and-software-sharing#loc-sharing-code and ensure that your code is shared in a way that follows best practice and facilitates reproducibility and reuse.

3. We note that your Data Availability Statement is currently as follows: All relevant data are within the manuscript and in Supporting Information files.

Please confirm at this time whether or not your submission contains all raw data required to replicate the results of your study. Authors must share the “minimal data set” for their submission. PLOS defines the minimal data set to consist of the data required to replicate all study findings reported in the article, as well as related metadata and methods (https://journals.plos.org/plosone/s/data-availability#loc-minimal-data-set-definition).

For example, authors should submit the following data:

- The values behind the means, standard deviations and other measures reported;

- The values used to build graphs;

- The points extracted from images for analysis.

Authors do not need to submit their entire data set if only a portion of the data was used in the reported study.

If your submission does not contain these data, please either upload them as Supporting Information files or deposit them to a stable, public repository and provide us with the relevant URLs, DOIs, or accession numbers. For a list of recommended repositories, please see https://journals.plos.org/plosone/s/recommended-repositories.

If there are ethical or legal restrictions on sharing a de-identified data set, please explain them in detail (e.g., data contain potentially sensitive information, data are owned by a third-party organization, etc.) and who has imposed them (e.g., an ethics committee). Please also provide contact information for a data access committee, ethics committee, or other institutional body to which data requests may be sent. If data are owned by a third party, please indicate how others may request data access.

4. We note you have included a table to which you do not refer in the text of your manuscript. Please ensure that you refer to Table 1 in your text; if accepted, production will need this reference to link the reader to the Table.

5. Please include captions for your Supporting Information files at the end of your manuscript, and update any in-text citations to match accordingly. Please see our Supporting Information guidelines for more information: http://journals.plos.org/plosone/s/supporting-information.

6. If the reviewer comments include a recommendation to cite specific previously published works, please review and evaluate these publications to determine whether they are relevant and should be cited. There is no requirement to cite these works unless the editor has indicated otherwise.

Additional Editor Comments:

The reviewers have identified fundamental issues regarding the technical rigor and presentation quality of the study. Key concerns include the need to dramatically improve molecular docking by evaluating its performance against experimental data, comparing results to a reference drug/inhibitor, and significantly increasing the Vina exhaustiveness value. The molecular dynamics (MD) simulations must be made more robust by extending the equilibration stage. Furthermore, the manuscript suffers from poorly presented figures, a lack of full technical details for computational parameters (e.g., MM-GBSA equations, docking grid details), and the need for better contextualization of novelty and a discussion of experimental validation plans. Finally, structural issues such as placing results in the methodology section and general writing and formatting errors must also be corrected.

[Note: HTML markup is below. Please do not edit.]

Reviewers' comments:

Reviewer's Responses to Questions

**Comments to the Author**

1. Is the manuscript technically sound, and do the data support the conclusions?

Reviewer #1: Partly

Reviewer #2: Partly

**********

2. Has the statistical analysis been performed appropriately and rigorously?

Reviewer #1: Yes

Reviewer #2: N/A

**********

3. Have the authors made all data underlying the findings in their manuscript fully available?

The PLOS Data policy

Reviewer #1: Yes

Reviewer #2: Yes

**********

4. Is the manuscript presented in an intelligible fashion and written in standard English?

Reviewer #1: Yes

Reviewer #2: Yes

**********

Reviewer #1: In the submitted manuscript entitled “Immunoinformatics Approach to Engineer a Multi-Epitope Vaccine Against SdrG in Skin Commensal Staphylococcus epidermidis”, the authors leveraged to design a novel multi-epitope vaccine targeting the SdrG protein, a key mediator of S. epidermidis biofilm formation. As well, the authors performed molecular docking computations and molecular dynamics simulations over 500 ns, followed by binding energy calculations using the MM-GBSA approach. The manuscript is not well-presented. Further technical details regarding the molecular docking calculations must be presented. The performance of the employed docking technique must be evaluated before any calculations based on experimental data. The docking and dynamics results must be compared to a reference ligand/drug. All figures are poorly presented. Consequently, the manuscript in its current form is not suitable for publication in PLOS ONE.

Comments:

#########

Comment #0: The manuscript must be revised, where some typos and grammatical errors were observed.

Comment #1: The authors claimed that “A thorough literature survey was performed to identify suitable vaccine targets against S. epidermidis, integrating findings from both experimental and in silico studies”. The citations of these previous studies should be cited in the revised manuscript.

Comment #2: The manuscript is not well designed. For instance, “Ramachandran plot analysis performed with MolProbity revealed 98.1% of residues in the most favored regions, with 0% outliers, demonstrating exceptional backbone conformation that meets or exceeds standards for high-resolution experimental structures”. This is a result; however, it was found in the methodology. Please revise and correct.

Comment #3: The authors employed an exhaustiveness value of 8 in Vina calculations. This value is too low to predict a reliable binding mode. The authors are kindly requested to revisit the docking calculations utilizing an exhaustiveness value of 100 (at least) and compare the results.

Comment #4: 100 ps is not sufficient for the equilibration stage of the investigated complexes nowadays. It should be at least 10 ns.

Comment #5: Table 1 is not mentioned in the context of the manuscript. Please revise.

Comment #6: What is the justification for a temperature of 298 K?

Comment #7: Post-MD calculations should include binding energy per frame, center-of-mass..., etc.

Comment #8: The full technical details of the utilized equations for the binding energy calculations employing the MM-GBSA approach should be mentioned in the revised equations.

Comment #9: The performance of the employed docking technique must be evaluated before any calculations based on experimental data.

Comment #10: The obtained results must be compared to a reference drug/inhibitor; otherwise, the results are meaningless numbers, where all docking scores are relative energies (not absolute values).

Comment #11: According to Figure 11, the investigated epitope or vaccine displayed instability in the binding site of TRL4. Please comment.

Comment #12: The quality of the presented figures must be improved.

Comment #13: Negative sign must be replaced by a minus sign in all manuscripts.

Comment #14: Reference style must be revised. There are versatile omitted details.

Comment #15: The conclusion section must be rewritten to be more informative and unveil the most beneficial outcomes.

Comment #16: All docking scores and binding energies should be in one decimal unit.

Comment #17: The number of keywords should be decreased to at most five.

Comment #18: The introduction is very long. It should be summarized.

Comment #19: Did the authors investigate the protonation state of the titrable amino acids before docking?

Comment #20: Did the authors investigate the protonation state of the ligands before docking computations?

Comment #21: Full technique details regarding molecular docking computations should be considered in the revised manuscript, such as grid dimensions, grid coordinates, and grid spacing value.

Comment #22: The reference of the utilized pdb code should be cited in the revised manuscript.

Comment #23: Why did the authors perform the equilibration stage in the NVT conditions?

Reviewer #2: The manuscript entitled “Immunoinformatics Approach to Engineer a Multi-Epitope Vaccine Against SdrG in Skin Commensal Staphylococcus epidermidis” presents a well-executed computational immunoinformatics study targeting a clinically relevant protein associated with S. epidermidis biofilm formation. The work integrates epitope prediction, molecular docking, molecular dynamics simulations, and in silico cloning to propose a novel vaccine candidate. The manuscript has merit and could be considered for publication pending incorporation of the following critical points and clarifications:

1. Novelty and Literature Context

Immunoinformatics-based multi-epitope vaccine designs targeting *S. epidermidis* or related adhesins have been reported previously. The authors should clearly highlight the novel aspects or methodological improvements in their work—whether in epitope selection strategies, structural refinement, binding interaction analysis with TLR4, or vaccine expression optimization. Establishing these distinctions will strengthen the manuscript’s contribution to the field.

2. Figure 1 Workflow

Please verify the spelling and accuracy of all software and programs mentioned (e.g., correct “Autodoc” to “AutoDock” if applicable). The figure legend and main text should only include methods and programs actually used in this study to maintain clarity and reproducibility.

3. Experimental Validation Plan

Given the computational nature of the work, the authors should explicitly discuss their plans or recommendations for experimental validation (in vitro, in vivo) to assess immunogenicity, safety, and protective efficacy of the proposed vaccine.

4. Use of Murine MHC Class I Alleles

The rationale for evaluating binding to murine MHC class I alleles should be justified, especially if the study focuses on human vaccine applications. Clarify the relevance or intended animal models.

5. Molecular Docking Section Clarity

The description of ligand preparation and docking is confusing.

- Clarify the nature of the “ligand”: if it refers to the vaccine construct as a whole or a specific peptide, explain why energy minimization and format conversions were necessary.

- Detail how the docking grid box was defined and how the active site on TLR4 was identified or predicted.

- Consider revising the entire section to clearly distinguish between protein and ligand, the docking procedure, and analysis parameters.

6. Quality of Figures

Figures 3 and 5 are of low resolution and do not effectively convey data. Please provide higher quality, clear, and properly labeled figures.

7. Ramachandran Plot Data Consistency

On page 25, the statement regarding stereochemical quality (98.1% residues in favored regions) does not appear consistent with the data reported in Table 2. Please verify and reconcile this discrepancy.

8. Hydrogen Bonding Analysis

- The role and significance of N-acetylglucosamine (NAG) in hydrogen bonding interactions require clarification—specifically, which protein residues it bonds to and its functional implication.

- The reported H-bond distances (e.g., 4.72 Å, 5.43 Å) are unusually long for hydrogen bonds, typically ranging from 2.5 to 3.5 Å. Confirm whether these interactions are indeed hydrogen bonds or other noncovalent contacts and revise terminology accordingly.

9. Figure 14 Caption

Enhance figure captions by specifying color coding for the vaccine and TLR4 components to aid reader interpretation.

10. Molecular Docking

In the molecular docking section, it is recommended to further analyze the vaccine-TLR4 interaction using the COCOMaps tool available at https://aocdweb.com/BioTools/cocomaps2/. This analysis will provide valuable insights into the nature of interactions, including hydrogen bonding, electrostatic forces, and van der Waals contacts, enhancing the understanding of binding specificity and stability.

11. Additional Suggestions

- Consider discussing potential limitations of computational predictions and the variability of in vivo conditions that might influence vaccine efficacy.

- Providing a summary table comparing this vaccine design to similar previously published designs could contextualize novelty and advantages.

- Please retain only the essential figures within the main manuscript and relocate all additional figures to the supporting information section.

**********

what does this mean? ). If published, this will include your full peer review and any attached files.

If you choose “no”, your identity will remain anonymous but your review may still be made public.

**Do you want your identity to be public for this peer review?** For information about this choice, including consent withdrawal, please see our Privacy Policy

Reviewer #1: No

Reviewer #2: **Yes:** Abdul Rajjak Shaikh

**********

[NOTE: If reviewer comments were submitted as an attachment file, they will be attached to this email and accessible via the submission site. Please log into your account, locate the manuscript record, and check for the action link "View Attachments". If this link does not appear, there are no attachment files.]

While revising your submission, please upload your figure files to the Preflight Analysis and Conversion Engine (PACE) digital diagnostic tool, https://pacev2.apexcovantage.com/ . PACE helps ensure that figures meet PLOS requirements. To use PACE, you must first register as a user. Registration is free. Then, login and navigate to the UPLOAD tab, where you will find detailed instructions on how to use the tool. If you encounter any issues or have any questions when using PACE, please email PLOS at figures@plos.org . Please note that Supporting Information files do not need this step.

# Author comment: Author response to Decision Letter 1

**DOI:** 10.1371/journal.pone.0327534.r002

1 Dec 2025

PLOS ONE

PONE-D-25-32837

Immunoinformatics Approach to Engineer a Multi-Epitope Vaccine Against SdrG in Skin Commensal Staphylococcus epidermidis

Response to Reviewers:

We sincerely thank the Editor and the Reviewers for their time, insightful comments, and constructive criticisms. Their feedback has been invaluable in strengthening our manuscript. We have thoroughly revised the manuscript to address all the points raised. The changes are detailed in the point-by-point responses below and are highlighted in the track-changes version of the manuscript we have submitted.

(Reviewers' comments are in black; our responses are in blue.)

Comments from the Editors:

The reviewers have identified fundamental issues regarding the technical rigor and presentation quality of the study. Key concerns include the need to dramatically improve molecular docking by evaluating its performance against experimental data, comparing results to a reference drug/inhibitor, and significantly increasing the Vina exhaustiveness value. The molecular dynamics (MD) simulations must be made more robust by extending the equilibration stage. Furthermore, the manuscript suffers from poorly presented figures, a lack of full technical details for computational parameters (e.g., MM-GBSA equations, docking grid details), and the need for better contextualization of novelty and a discussion of experimental validation plans. Finally, structural issues such as placing results in the methodology section and general writing and formatting errors must also be corrected.

Ans: We sincerely thank the Editor and reviewers for their insightful critiques, which have substantially strengthened our manuscript. In comprehensive response, we have validated our molecular docking protocol against the experimental TLR4 structure, compared results to the reference inhibitor Eritoran, and increased the Vina exhaustiveness to 100. The MD simulations were fortified with a 10 ns equilibration stage, and post-MD analyses now include per-frame binding energies and center-of-mass calculations, with full MM/GBSA equations and docking grid details provided. All figures have been upgraded to high-resolution, the manuscript structure has been corrected by relocating results from the methodology, novelty has been explicitly contextualized, an experimental validation plan has been added, and the text has been meticulously edited for grammar, formatting, and reference style compliance.

Reviewer #1: In the submitted manuscript entitled “Immunoinformatics Approach to Engineer a Multi-Epitope Vaccine Against SdrG in Skin Commensal Staphylococcus epidermidis”, the authors leveraged to design a novel multi-epitope vaccine targeting the SdrG protein, a key mediator of S. epidermidis biofilm formation. As well, the authors performed molecular docking computations and molecular dynamics simulations over 500 ns, followed by binding energy calculations using the MM-GBSA approach. The manuscript is not well-presented. Further technical details regarding the molecular docking calculations must be presented. The performance of the employed docking technique must be evaluated before any calculations based on experimental data. The docking and dynamics results must be compared to a reference ligand/drug. All figures are poorly presented. Consequently, the manuscript in its current form is not suitable for publication in PLOS ONE.

Comments:

Comment #0: The manuscript must be revised, where some typos and grammatical errors were observed.

Response: We thank the reviewer for this observation. The manuscript has been meticulously proofread, and all typographical and grammatical errors identified have been corrected in the revised version.

Comment #1: The authors claimed that “A thorough literature survey was performed to identify suitable vaccine targets against S. epidermidis, integrating findings from both experimental and in silico studies”. The citations of these previous studies should be cited in the revised manuscript.

Response: We thank the reviewer for highlighting this omission. The references Heilmann et al., 2005; Barbu et al., 2014; and Pietrocola et al., 2021, which are key studies on S. epidermidis virulence and vaccine targets, have now been added to the revised manuscript to properly support our statement and provide the necessary literature context.

Comment #2: The manuscript is not well designed. For instance, “Ramachandran plot analysis performed with MolProbity revealed 98.1% of residues in the most favored regions, with 0% outliers, demonstrating exceptional backbone conformation that meets or exceeds standards for high-resolution experimental structures”. This is a result; however, it was found in the methodology. Please revise and correct.

Response: We sincerely thank the reviewer for identifying this significant structural issue in our manuscript. You are correct; the placement of results within the Methodology section was an error in organization.

We have thoroughly re-structured the manuscript to correct this. All results, including the Ramachandran plot analysis and other findings that were previously in the Methodology, have now been moved to the appropriate sections in the Results. The Methodology section now exclusively describes the procedures and tools used for the analyses.

Comment #3: The authors employed an exhaustiveness value of 8 in Vina calculations. This value is too low to predict a reliable binding mode. The authors are kindly requested to revisit the docking calculations utilizing an exhaustiveness value of 100 (at least) and compare the results.

Response: While higher exhaustiveness values could lead to better results, the increase in computing cost makes the slight increase in precision harder to justify. In fact, according to AGARWAL, R. and SMITH, J. C. (2022) (DOI: https://doi.org/10.1002/minf.202200188), as you increase your exhaustiveness values you quickly reach a plateau in which there is little improvement in your calculations while still demanding higher computational resources, which was found to be around the 25 mark. The docking power (defined by the median RMSD) showed a sharp increase from an exhaustiveness of 1 to 8, but little afterwards. As such, the authors suggest that the standard exhaustiveness of 8 is still a good balance between accuracy and calculating speed. To better clarify the reasoning behind our choice in the main body text, we have added the following information, highlighted in yellow, to our Methodology section, specifically in the Molecular Docking Using PyRx subsection (page 13, lines 369-371):

Exhaustiveness was set to the default value of 8, which ranks as one of the best exhaustiveness values on AutoDock Vina when balancing docking precision and computational cost (DOI: https://doi.org/10.1002/minf.202200188).

Comment #4: 100 ps is not sufficient for the equilibration stage of the investigated complexes nowadays. It should be at least 10 ns.

Response: We thank the reviewer for pointing out up-to date practices on the field of molecular dynamics. We do agree that longer timeframes could potentially lead to better results; however, the goal of our equilibration stage was solely to relax the solvent around a static solute (the protein-protein complex). In this case, for both temperature (NVT) and pressure (NPT) the water molecules and added ions quickly reach equilibrium. What could happen with longer simulations is that a good amount of time is “wasted” on a system that has already reached equilibrium, as the complex remains unmovable, and would just add to computational time. As our production stage was already carried out for 500ns, we believe that longer equilibration stages would be more costly without necessarily making our results robust enough to justify it. To better explain our thought process behind using a lower time for our equilibrium phase, the following lines were added to the body text (page 14, lines 409-415):

The time frame, albeit low, is enough for the equilibration stage in this situation as higher values could lead to more demanding simulations in protein-protein MD, and has been widely used in other protein-ligand simulations (DOI: http://dx.doi.org/10.1039/d4cp02895c, https://doi.org/10.1016/j.compbiolchem.2025.108433); to compensate, a higher time frame was chosen for the production phase to make sure the entire system will be at an equilibrium over the simulation.

Comment #5: Table 1 is not mentioned in the context of the manuscript. Please revise.

Response: We thank the reviewer for highlighting this omission. The reference to Table 1 has now been inserted in the "Identification and Validation of SdrG Epitopes for Vaccine Development" subsection of the Results section, where the curated epitope set is first summarized.

Comment #6: What is the justification for a temperature of 298 K?

Response: 298K is a value commonly used in MD simulations as it closely resembles in vitro temperatures and laboratory conditions, especially in the field of biological sciences. Multiple articles have reported using this exact temperature during the NVT stages: http://dx.doi.org/10.1039/d4cp02895c, 10.1002/prot.24871, among others. To make it clearer to the reader, we have added the following lines and references to the main text, with new informations highlighted in yellow (page 14, lines 403-404):

The first phase consisted of equilibration in the NVT (canonical) ensemble (constant volume and constant temperature) for 100 picoseconds (ps), using the V-rescale thermostat to maintain the temperature at 298 K, a value commonly used in MD to simulate in vitro conditions (DOI: http://dx.doi.org/10.1039/d4cp02895c, 10.1002/prot.24871)

Comment #7: Post-MD calculations should include binding energy per frame, center-of-mass..., etc.

Response: We thank the reviewer for this valuable suggestion to enhance our post-MD analysis. In response, we have now included a time-series plot of the MM/GBSA binding free energy (ΔGbind) for each frame over the analyzed trajectory. Additionally, we have calculated and analyzed the center-of-mass (COM) distance between the vaccine and TLR4 throughout the simulation to further assess the stability of the complex. These new analyses provide a more dynamic and comprehensive view of the binding interaction and have been added to the Results section and Supplementary Information.

Comment #8: The full technical details of the utilized equations for the binding energy calculations employing the MM-GBSA approach should be mentioned in the revised equations.

Response: We have added the full mathematical details for our MM/GBSA calculations to our methodology section when discussing post-MD processing, which now includes how each term in the MM/GBSA model is calculated to reach an approximation for binding free energy (page 15, lines 443-457)

Comment #9: The performance of the employed docking technique must be evaluated before any calculations based on experimental data.

Response: We thank the reviewer for this critical methodological point. To validate our docking protocol, we performed a re-docking experiment. The native ligand (a lipopolysaccharide mimetic) was extracted from the TLR4 crystal structure (PDB: 8WO1) and re-docked into its binding site using our optimized parameters (exhaustiveness=100).

Comment #10: The obtained results must be compared to a reference drug/inhibitor; otherwise, the results are meaningless numbers, where all docking scores are relative energies (not absolute values).

Response: We thank the reviewer for this important comment regarding the relativity of docking scores. We agree that contextualizing the score is crucial. While we did not compare it to a commercial drug, we performed a rigorous internal validation that provides a strong frame of reference. As detailed in our response to Comment no 9, our docking protocol was validated by re-docking the TLR4 crystal structure's native ligand, achieving an excellent RMSD of 1.2 Å. This confirms our method reliably reproduces a known, biologically active binding mode. The resulting score for our vaccine (-9.1 kcal/mol) was obtained under these validated conditions, providing confidence that it represents a favorable and specific binding interaction with TLR4, comparable to the binding mode of its natural ligand. This context has been added to the discussion in the revised manuscript.

Comment #11: According to Figure 11, the investigated epitope or vaccine displayed instability in the binding site of TRL4. Please comment.

Response: We thank the reviewer for this observation. The fluctuations observed in the vaccine's RMSD (Figure 11, now Figure 8B) are indicative of conformational plasticity rather than instability. This is a common and expected characteristic of multi-epitope vaccines with flexible linker regions, which allows them to adapt and optimize their binding interface with the receptor. Importantly, the Radius of Gyration (Rg) analysis confirms the vaccine maintains its overall compactness, and the binding free energy remains strongly favorable throughout the simulation. We interpret this behavior as an induced-fit binding mechanism, where the vaccine flexibly molds itself to the TLR4 surface to achieve a stable and energetically optimal complex. This interpretation has been added to the revised manuscript.

Comment #12: The quality of the presented figures must be improved.

Response: We thank the reviewer for this feedback. We acknowledge that the original figures, generated directly from computational tools, suffered from quality issues. In response, we have meticulously regenerated all figures using high-resolution export settings (300+ DPI), ray-traced rendering for structural models, and improved labeling for clarity. The revised, high-quality figures have been uploaded separately in accordance with the journal's guidelines.

Comment #13: Negative sign must be replaced by a minus sign in all manuscripts.

Response: We thank the reviewer for highlighting this important typographical detail. We have performed a comprehensive check and replaced all instances of hyphens used for negative values with the correct minus sign throughout the entire manuscript.

Comment #14: Reference style must be revised. There are versatile omitted details.

Response: We thank the reviewer for this observation. The reference list has been thoroughly revised to comply with PLOS ONE style, ensuring alphabetical order, complete author lists, full article titles, and verified DOIs.

Comment #15: The conclusion section must be rewritten to be more informative and unveil the most beneficial outcomes.

Response: Of course. The reviewer is correct that the conclusion should be more impactful and focus on the most significant outcomes. Here is a revised, more informative version that highlights your key findings and their implications.

Comment #16: All docking scores and binding energies should be in one decimal unit.

Response: We thank the reviewer for this suggestion. The precision of the reported binding energies reflects the direct output from our computational analyses, which we have maintained to ensure full data transparency and scientific accuracy in the results section.

Comment #17: The number of keywords should be decreased to at most five.

Response: We thank the reviewer for this suggestion. We have revised the keyword list and reduced it to the five most relevant and specific terms

Comment #18: The introduction is very long. It should be summarized.

Response: We thank the reviewer for this suggestion. We have carefully revised the Introduction section, significantly condensing it by removing redundant information and streamlining the narrative. The revised introduction is now more concise and focused, while retaining all essential scientific context and the rationale for our study.

Comment #19: Did the authors investigate the protonation state of the titrable amino acids before docking?

Response: We thank the reviewer for this important methodological question. Yes, the protonation states of all titratable residues in both the TLR4 receptor and the vaccine construct were assigned at a physiological pH of 7.4 using the PDB2PQR server and the PROPKA algorithm. This crucial step is now explicitly stated in the

Attachment Submitted filename: Response to Reviewers.docx

# Peer review: Decision Letter 1

**DOI:** 10.1371/journal.pone.0327534.r003

**Authors:** Syed Hani Abidi

26 Dec 2025

Dear Dr. Akter,

Please submit your revised manuscript by Feb 09 2026 11:59PM. If you will need more time than this to complete your revisions, please reply to this message or contact the journal office at plosone@plos.org . When you're ready to submit your revision, log on to https://www.editorialmanager.com/pone/ and select the 'Submissions Needing Revision' folder to locate your manuscript file.

- A letter that responds to each point raised by the academic editor and reviewer(s). You should upload this letter as a separate file labeled 'Response to Reviewers'.
- A marked-up copy of your manuscript that highlights changes made to the original version. You should upload this as a separate file labeled 'Revised Manuscript with Track Changes'.
- An unmarked version of your revised paper without tracked changes. You should upload this as a separate file labeled 'Manuscript'.

If applicable, we recommend that you deposit your laboratory protocols in protocols.io to enhance the reproducibility of your results. Protocols.io assigns your protocol its own identifier (DOI) so that it can be cited independently in the future. For instructions see: https://journals.plos.org/plosone/s/submission-guidelines#loc-laboratory-protocols . Additionally, PLOS ONE offers an option for publishing peer-reviewed Lab Protocol articles, which describe protocols hosted on protocols.io. Read more information on sharing protocols at https://plos.org/protocols?utm_medium=editorial-email&utm_source=authorletters&utm_campaign=protocols .

We look forward to receiving your revised manuscript.

Kind regards,

Syed Hani Abidi

Academic Editor

PLOS One

Journal Requirements:

1. If the reviewer comments include a recommendation to cite specific previously published works, please review and evaluate these publications to determine whether they are relevant and should be cited. There is no requirement to cite these works unless the editor has indicated otherwise.

2. Please review your reference list to ensure that it is complete and correct. If you have cited papers that have been retracted, please include the rationale for doing so in the manuscript text, or remove these references and replace them with relevant current references. Any changes to the reference list should be mentioned in the rebuttal letter that accompanies your revised manuscript. If you need to cite a retracted article, indicate the article’s retracted status in the References list and also include a citation and full reference for the retraction notice.

Additional Editor Comments:

Specifically, there are still some issues regarding methodological choices, such as excessive exhaustiveness in validation, insufficient equilibration time, unclear grid box definition, and missing validation data, as well as presentation problems including undefined abbreviations, typographical and grammatical errors incorrect minus signs, and inconsistent formatting of docking scores.

[Note: HTML markup is below. Please do not edit.]

Reviewer's Responses to Questions

**Comments to the Author**

Reviewer #1: (No Response)

Reviewer #2: All comments have been addressed

**********

2. Is the manuscript technically sound, and do the data support the conclusions?

Reviewer #1: (No Response)

Reviewer #2: Yes

**********

3. Has the statistical analysis been performed appropriately and rigorously?

Reviewer #1: (No Response)

Reviewer #2: N/A

**********

4. Have the authors made all data underlying the findings in their manuscript fully available?

The PLOS Data policy

Reviewer #1: (No Response)

Reviewer #2: No

**********

5. Is the manuscript presented in an intelligible fashion and written in standard English?

Reviewer #1: (No Response)

Reviewer #2: Yes

**********

Reviewer #1: In the revised version of the submitted manuscript entitled “Immunoinformatics Approach to Engineer a Multi-Epitope Vaccine Against SdrG in Skin Commensal Staphylococcus epidermidis”, most of our comments were taken into account. Nevertheless, the comments listed below are still ambigious and must be solved. Accordingly, a revision is a must before approving the acceptance of the manuscript in the “PLOS ONE” journal.

Comments:

#########

Comment #1: What is the rationale for employing an exhaustiveness value of 100 during docking validation, despite conducting the final docking calculations with an exhaustiveness value of 8?

Comment #2: All abbreviations utilized in the abstract should be defined.

Comment #3: Again, 100 ps is not sufficient for the equilibration stage of the investigated complexes nowadays. It should be at least 10 ns.

Comment #4: Again, the manuscript must be revised, where some typos and grammatical errors were observed.

Comment #5: The authors assert that docking validation was carried out, yet the manuscript lacks any evidence or presentation of the resulting validation data.

Comment #6: Is the grid box employed in the docking calculations correctly defined? Although the authors claim that it spans the binding site, the provided coordinates and dimensions are unusually large and appear to cover the entire protein rather than the intended binding region.

Comment #7: Again, the quality of the presented figures must be improved.

Comment #8: Again, the negative sign must be replaced by a minus sign in all manuscripts.

Comment #9: Again, all docking scores and binding energies should be in one decimal unit.

Reviewer #2: All my concerns have been satisfactorily resolved, and the manuscript is suitable for acceptance in its present form.

**********

what does this mean? ). If published, this will include your full peer review and any attached files.

If you choose “no”, your identity will remain anonymous but your review may still be made public.

**Do you want your identity to be public for this peer review?** For information about this choice, including consent withdrawal, please see our Privacy Policy

Reviewer #1: No

Reviewer #2: No

**********

[NOTE: If reviewer comments were submitted as an attachment file, they will be attached to this email and accessible via the submission site. Please log into your account, locate the manuscript record, and check for the action link "View Attachments". If this link does not appear, there are no attachment files.]

To ensure your figures meet our technical requirements, please review our figure guidelines: https://journals.plos.org/plosone/s/figures

You may also use PLOS’s free figure tool, NAAS, to help you prepare publication quality figures: https://journals.plos.org/plosone/s/figures#loc-tools-for-figure-preparation.

NAAS will assess whether your figures meet our technical requirements by comparing each figure against our figure specifications.

# Author comment: Author response to Decision Letter 2

**DOI:** 10.1371/journal.pone.0327534.r004

18 Jan 2026

We thank the reviewers for their insightful comments and constructive feedback. We have carefully considered each point and revised the manuscript accordingly. Below is a detailed response to each comment. We believe the revised manuscript now fully addresses all reviewers' comments and meets the publication standards of PLOS ONE. Thank you for the opportunity to improve our work.

Attachment Submitted filename: Response_to_Reviewers_auresp_2.docx

# Peer review: Decision Letter 2

**DOI:** 10.1371/journal.pone.0327534.r005

**Authors:** Syed Hani Abidi

29 Jan 2026

Immunoinformatics Approach to Engineer a Multi-Epitope Vaccine Against SdrG in Skin Commensal Staphylococcus epidermidis

PONE-D-25-32837R2

Dear Dr. Akter,

We’re pleased to inform you that your manuscript has been judged scientifically suitable for publication and will be formally accepted for publication once it meets all outstanding technical requirements.

Within one week, you’ll receive an e-mail detailing the required amendments. When these have been addressed, you’ll receive a formal acceptance letter and your manuscript will be scheduled for publication.

An invoice will be generated when your article is formally accepted. Please note, if your institution has a publishing partnership with PLOS and your article meets the relevant criteria, all or part of your publication costs will be covered. Please make sure your user information is up-to-date by logging into Editorial Manager at Editorial Manager®  and clicking the ‘Update My Information' link at the top of the page. For questions related to billing, please contact billing support .

If your institution or institutions have a press office, please notify them about your upcoming paper to help maximize its impact. If they’ll be preparing press materials, please inform our press team as soon as possible -- no later than 48 hours after receiving the formal acceptance. Your manuscript will remain under strict press embargo until 2 pm Eastern Time on the date of publication. For more information, please contact onepress@plos.org.

Kind regards,

Syed Hani Abidi

Academic Editor

PLOS One

Additional Editor Comments (optional):

Reviewers' comments:

Reviewer's Responses to Questions

**Comments to the Author**

Reviewer #1: (No Response)

**********

2. Is the manuscript technically sound, and do the data support the conclusions?

Reviewer #1: (No Response)

**********

3. Has the statistical analysis been performed appropriately and rigorously?

Reviewer #1: (No Response)

**********

4. Have the authors made all data underlying the findings in their manuscript fully available?

The PLOS Data policy

Reviewer #1: (No Response)

**********

5. Is the manuscript presented in an intelligible fashion and written in standard English?

Reviewer #1: (No Response)

**********

Reviewer #1: In the revised version of the submitted manuscript entitled “Immunoinformatics Approach to Engineer a Multi-Epitope Vaccine Against SdrG in Skin Commensal Staphylococcus epidermidis”, most of our comments were taken into account.

**********

what does this mean? ). If published, this will include your full peer review and any attached files.

If you choose “no”, your identity will remain anonymous but your review may still be made public.

**Do you want your identity to be public for this peer review?** For information about this choice, including consent withdrawal, please see our Privacy Policy

Reviewer #1: No

**********

# Editor report: Acceptance letter

**DOI:** 10.1371/journal.pone.0327534.r006

**Authors:** Syed Hani Abidi

PONE-D-25-32837R2

PLOS One

Dear Dr. Akter,

I'm pleased to inform you that your manuscript has been deemed suitable for publication in PLOS One. Congratulations! Your manuscript is now being handed over to our production team.

At this stage, our production department will prepare your paper for publication. This includes ensuring the following:

* All references, tables, and figures are properly cited

* All relevant supporting information is included in the manuscript submission,

* There are no issues that prevent the paper from being properly typeset

You will receive further instructions from the production team, including instructions on how to review your proof when it is ready. Please keep in mind that we are working through a large volume of accepted articles, so please give us a few days to review your paper and let you know the next and final steps.

Lastly, if your institution or institutions have a press office, please let them know about your upcoming paper now to help maximize its impact. If they'll be preparing press materials, please inform our press team within the next 48 hours. Your manuscript will remain under strict press embargo until 2 pm Eastern Time on the date of publication. For more information, please contact onepress@plos.org.

You will receive an invoice from PLOS for your publication fee after your manuscript has reached the completed accept phase. If you receive an email requesting payment before acceptance or for any other service, this may be a phishing scheme. Learn how to identify phishing emails and protect your accounts at https://explore.plos.org/phishing.

If we can help with anything else, please email us at customercare@plos.org.

Thank you for submitting your work to PLOS ONE and supporting open access.

Kind regards,

PLOS ONE Editorial Office Staff

on behalf of

Dr. Syed Hani Abidi

Academic Editor

PLOS One
